# Lack of Chemokine Signaling through CXCR5 Causes Increased Mortality, Ventricular Dilatation and Deranged Matrix during Cardiac Pressure Overload

**DOI:** 10.1371/journal.pone.0018668

**Published:** 2011-04-18

**Authors:** Anne Waehre, Bente Halvorsen, Arne Yndestad, Cathrine Husberg, Ivar Sjaastad, Ståle Nygård, Christen P. Dahl, M. Shakil Ahmed, Alexandra V. Finsen, Henrik Reims, William E. Louch, Denise Hilfiker-Kleiner, Leif E. Vinge, Borghild Roald, Håvard Attramadal, Martin Lipp, Lars Gullestad, Pål Aukrust, Geir Christensen

**Affiliations:** 1 Institute for Experimental Medical Research, Oslo University Hospital Ullevål, Oslo, Norway; 2 Research Institute for Internal Medicine, Oslo University Hospital Rikshospitalet, Oslo, Norway; 3 Institute for Surgical Research, Oslo University Hospital Rikshospitalet, Oslo, Norway; 4 Department of Cardiology, Oslo University Hospital Rikshospitalet, Oslo, Norway; 5 Department of Cardiology, Oslo University Hospital Ullevål, Oslo, Norway; 6 Department of Pathology, Oslo University Hospital Ullevål, Oslo, Norway; 7 Department of Cardiology and Angiology, Hanover Medical School, Hanover, Germany; 8 Department of Molecular Tumor Genetics and Immunogenetics, Max-Delbrück-Center for Molecular Medicine, Berlin, Germany; 9 Section of Clinical Immunology and Infectious Diseases, Oslo University Hospital Rikshospitalet, Oslo, Norway; 10 Faculty of Medicine, University of Oslo, Oslo, Norway; 11 Center for Heart Failure Research, University of Oslo, Oslo, Norway; 12 Bioinformatics Core Facility, Institute for Medical Informatics, Oslo University Hospital Rikshospitalet, Oslo, Norway; University of California Los Angeles, United States of America

## Abstract

**Rationale:**

Inflammatory mechanisms have been suggested to play a role in the development of heart failure (HF), but a role for chemokines is largely unknown. Based on their role in inflammation and matrix remodeling in other tissues, we hypothesized that CXCL13 and CXCR5 could be involved in cardiac remodeling during HF.

**Objective:**

We sought to analyze the role of the chemokine CXCL13 and its receptor CXCR5 in cardiac pathophysiology leading to HF.

**Methods and Results:**

Mice harboring a systemic knockout of the CXCR5 (CXCR5^−/−^) displayed increased mortality during a follow-up of 80 days after aortic banding (AB). Following three weeks of AB, CXCR5^−/−^ developed significant left ventricular (LV) dilatation compared to wild type (WT) mice. Microarray analysis revealed altered expression of several small leucine-rich proteoglycans (SLRPs) that bind to collagen and modulate fibril assembly. Protein levels of fibromodulin, decorin and lumican (all SLRPs) were significantly reduced in AB CXCR5^−/−^ compared to AB WT mice. Electron microscopy revealed loosely packed extracellular matrix with individual collagen fibers and small networks of proteoglycans in AB CXCR5^−/−^ mice. Addition of CXCL13 to cultured cardiac fibroblasts enhanced the expression of SLRPs. In patients with HF, we observed increased myocardial levels of CXCR5 and SLRPs, which was reversed following LV assist device treatment.

**Conclusions:**

Lack of CXCR5 leads to LV dilatation and increased mortality during pressure overload, possibly via lack of an increase in SLRPs. This study demonstrates a critical role of the chemokine CXCL13 and CXCR5 in survival and maintaining of cardiac structure upon pressure overload, by regulating proteoglycans essential for correct collagen assembly.

## Introduction

Heart failure (HF) is a disorder associated with low-grade immune activation and inflammation, as evidenced by elevated circulating and myocardial levels of inflammatory cytokines, including tumor necrosis factor (TNF)α, interleukin (IL)-1β and IL-18, and chemokines such as monocyte chemoattractant protein (MCP)-1 and fractalkine [1]–[5]. A range of experimental studies have also suggested a pathogenic role for several of these inflammatory mediators in the development and progression of HF [4], [6]–[8]. However, the role of inflammation in HF remains incompletely understood. Identification of the most important mediators of the inflammatory pathways involved in the pathogenesis of HF and their mechanism of action are issues that need to be further clarified.

While most chemokines have been linked to inflammatory processes in peripheral tissue, the homeostatic chemokines (i.e., CCL19, CCL21, and CXCL13) and their corresponding receptors (i.e., CCR7 for [CCL19 and CCL21] and CXCR5 for [CXCL13]) have been associated with development and maintenance of secondary lymphoid organs [9]–[12], as well as the entry of lymphocytes and dendritic cells to secondary lymphoid tissue [13]–[15]. Recently, however, reports have pointed to a broader role for these homeostatic chemokines, including modulation of inflammatory and anti-inflammatory responses in lymphoid and non-lymphoid tissue. Thus, while CXCL13 was known to dictate homing and motility of B cells in lymphoid tissue, more recent studies suggest that CXCL13 is involved in the formation of ectopic lymphoid tissue in chronic inflammation [16], [17]. This chemokine has also been linked to T cell [9], [18], [19] and monocyte activation [20] and apoptosis [21]. In line with its newly discovered role in the immune system, CXCL13 has been suggested to be involved in the pathogenesis of rheumatoid arthritis [22], Sjögren syndrome [23]–[25], inflammatory bowel disease [26], and multiple sclerosis [27]. CXCR5 is a G protein-coupled seven transmembrane receptor and belongs to the CXC chemokine receptor family [28]. Recently, CXCR5 has been found to be involved in remodeling of extracellular matrix (ECM) in various types of cancer, including colon [29] and prostate cancer [30]. However, the potential role for CXCL13 and CXCR5 in the pathogenesis of myocardial remodeling has not been studied.

Based on their potential role in inflammation and matrix remodeling, we hypothesized that CXCL13 and its receptor CXCR5 are involved in cardiac remodeling and development of HF. We examined this hypothesis by studying the cardiac morphology, function and molecular alterations in CXCR5 deficient (CXCR5^−/−^) mice exposed to left ventricular (LV) pressure overload induced by aortic banding (AB).

## Results

### Expression of CXCR5 and CXCL13 in murine hearts

**Figure 1:**
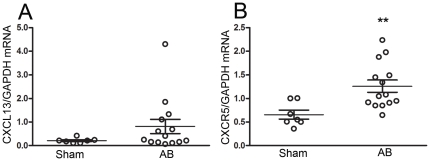
Myocardial gene expression of CXCL13 and CXCR5 in mice. Myocardial gene expression of (**A**) CXCL13 and (**B**) CXCR5 in wild type (WT) Sham (n = 7) and WT aorta banded (AB) (n = 14) group. The results are mean ± SEM. **p<0.01 *vs*. Sham group.

**Figure 2:**
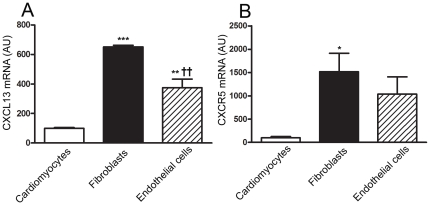
Gene expression of CXCL13 and CXCR5 in myocardial cells in mice. Gene expression of (**A**) CXCL13 and (**B**) CXCR5 in cardiomyocytes, fibroblasts and endothelial cells from wild type mice (n = 3). mRNA levels were assesssed by quantitaive real time PCR. AU = Arbitrary unit. The results are mean ± SEM. * p<0.05, **p<0.01, ***p<0.001 *vs*. cardiomyocytes. ††p <0.01 *vs.* fibroblasts.

We first examined if the CXCL13/CXCR5 dyad was regulated during AB in mice. Both CXCL13 and CXCR5 were expressed within the murine heart, and as shown in Fig. 1*A* and *B*, we found significantly enhanced myocardial expression of CXCR5, but not of CXCL13, in mice that underwent AB as compared with sham operated mice. Within the myocardium, we found mRNA expression of CXCL13 and CXCR5 in cardiomyocytes, fibroblasts and endothelial cells with the highest expression of both CXCR5 and CXCL13 in myocardial fibroblasts (Fig. 2*A, B*).

### Survival during LV pressure overload

**Figure 3:**
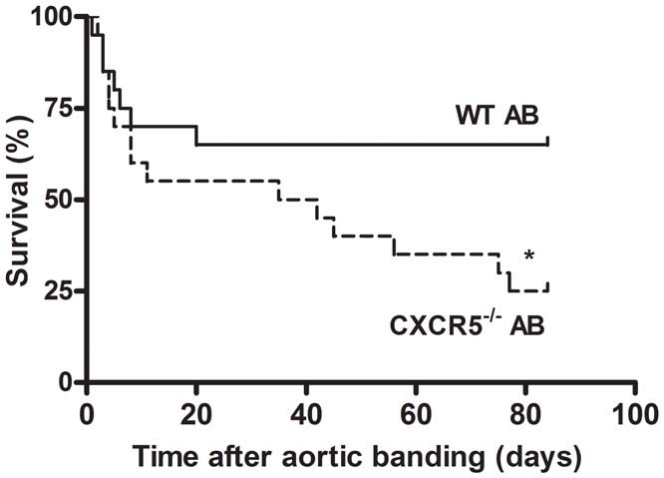
Kaplan-Meier survival curves of wild type (WT) (n = 20) and CXCR5^−/−^ mice (n = 20) after aortic banding (AB). Echocardiographic assessment showed comparable increases in flow velocities across the banded region of the aorta in WT and CXCR5^−/−^ twenty-one days after primary surgery. Differences in survival between WT and CXCR5^−/−^ mice were tested with the log-rank test. *p<0.05 *vs.* WT.

As depicted in Kaplan-Meier survival curves (Fig. 3), CXCR5^−/−^ mice exhibited significantly higher mortality rates than WT mice during an 80-day follow-up after AB induction. The differences in mortality emerged after 40 days of AB.

### Severe LV dilatation in CXCR5^−/−^ mice following pressure overload

**Figure 4:**
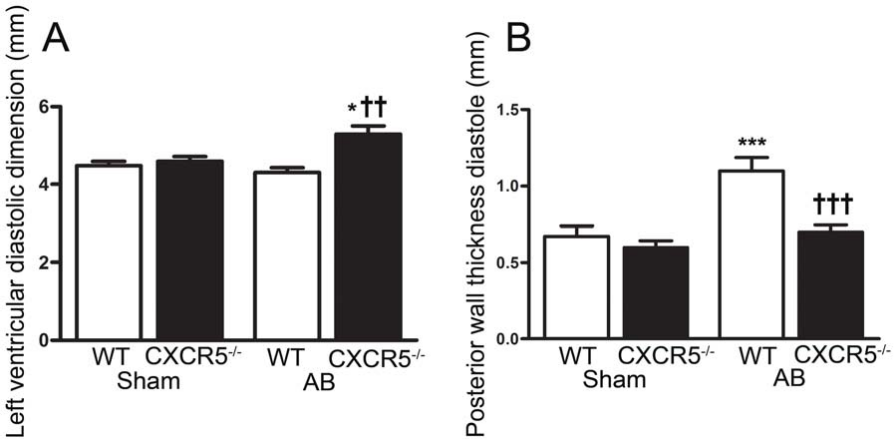
Left ventricular (LV) dilatation in CXCR5^−/−^ mice 3 weeks following aortic banding (AB). LV inner diameter (**A**) and thickness of the posterior wall (**B**) were measured in the wild type (WT) Sham (n = 6), CXCR5^−/−^ Sham (n = 6), WT AB (n = 6), and CXCR5^−/−^ AB (n = 6) groups. The results are mean ± SEM. *p<0.05 and ***p<0.001 *vs.* Sham groups; ††p<0.01 and †††p<0.001 *vs.* WT AB group.

To investigate a potential role for CXCR5 in cardiac remodeling and development of HF, we evaluated cardiac morphology and function in WT and CXCR5^−/−^ mice. Two-dimensional and M-mode echocardiography performed in non-operated WT (n = 6) and CXCR5^−/−^ (n = 6) mice showed no significant differences in LV function or dimensions (Table S1). Twenty-one days after AB, echocardiographic assessment showed comparable increases in flow velocities across the banded region of the aorta in the WT and CXCR5^−/−^ groups. However, LV fractional shortening was reduced by 65% in CXCR5^−/−^ mice, but only by 13% in WT (Table S1). The mean LV diastolic dimension was significantly larger and the LV posterior wall thickness was significantly lower in CXCR5^−/−^ mice compared to WT mice at the same time point (Fig. 4*A, B*).

### CXCR5^−/−^ mice show increased expression of hypertrophy maker genes in response to pressure overload

Despite similar increases in heart weight to tibial length (HW/TL) ratio in both genotypes after AB, CXCR5^−/−^ mice exhibited a more marked increase in expression of ANP (3.3-fold increase), BNP (2.3-fold increase) and β-MHC (2.5-fold increase) than WT (Fig. S1 *A–C*). The marked increase in ANP and BNP in CXCR5^−/−^ mice following AB might suggest increased myocardial wall stress in these mice.

### CXCR5^−/−^ mice exhibit major alterations in ECM in response to pressure overload

**Figure 5:**
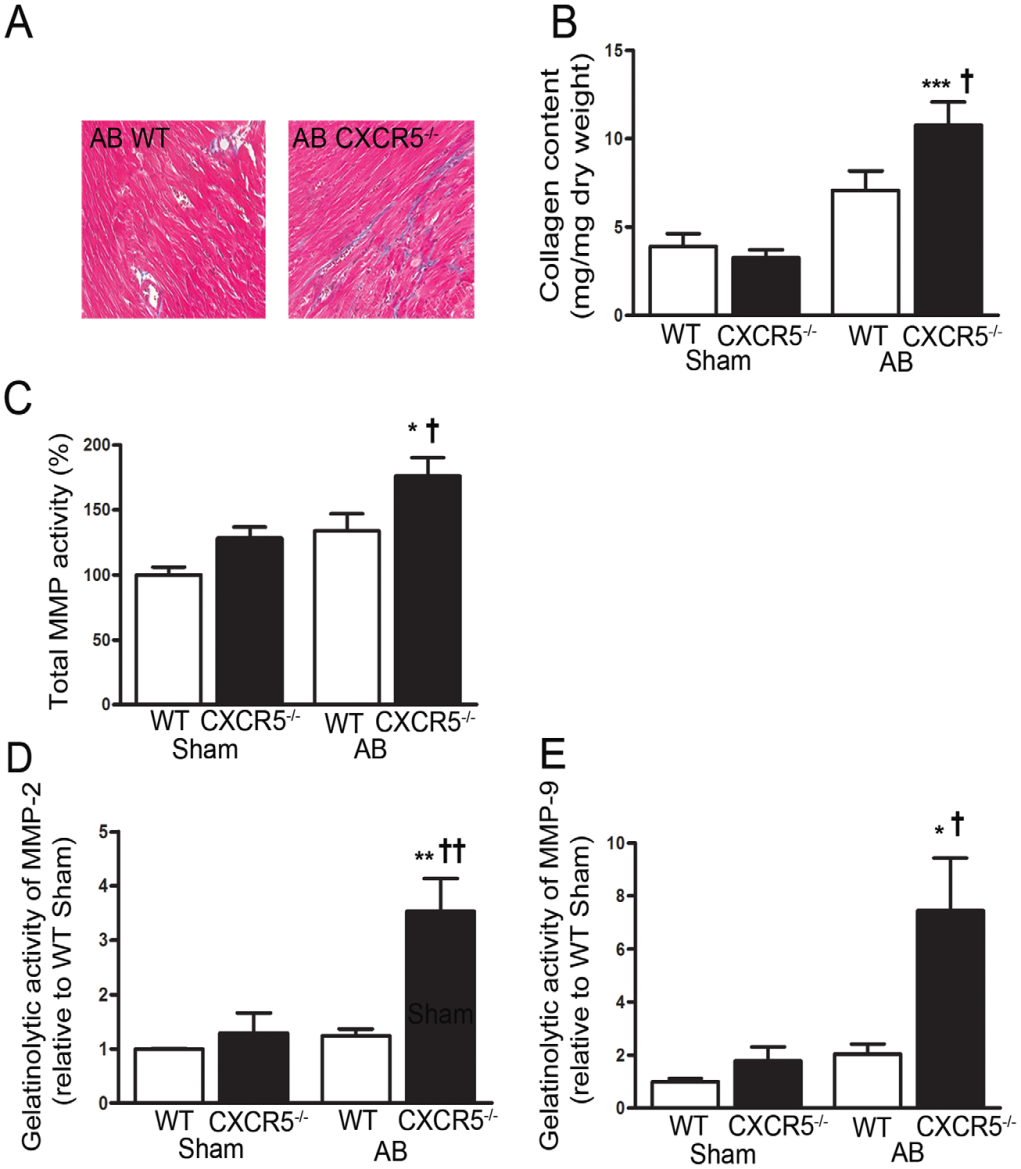
Extracellular matrix remodeling in wild type (WT) and CXCR5^−/−^ mice following aortic banding (AB). Collagen content in myocardium examined with (**A**) Masson trichrome staining in representative sections from AB WT and CXCR5^−/−^ mice and (**B**) by hydroxyproline measurement in WT Sham (n=13), CXCR5^−/−^ Sham (n=12), WT AB (n=13) and CXCR5^−/−^ AB (n=14) groups. (**C**) Total MMP activity in percentage of the WT Sham group (n=11) measured in the CXCR5^−/−^ Sham (n=13), WT AB (n=9), and CXCR5^−/−^ AB groups (n=9). (**D**) Gelatinolytic activity of MMP‐2 relative to the WT Sham group (n=5) measured in the CXCR5^−/−^ Sham (n=5), WT AB (n=5), and CXCR5^−/−^ AB groups (n=5) and of (*E*) MMP‐9 relative to the WT Sham group (n=6) measured in the CXCR5^−/−^ Sham (n=6), WT AB (n=8), and CXCR5^−/−^ AB groups (n=9). The results are mean ± SEM. *p<0.05, **p<0.01 and ***p<0.001 *vs.* Sham groups; †p<0.05 and ††p<0.01 *vs.* WT AB groups.

Since alterations in extracellular matrix (ECM) might be responsible for LV dilatation, we examined the quality and composition of the ECM following AB. CXCR5^−/−^ mice exhibited increased myocardial collagen content following AB as compared with WT mice, as illustrated by both Masson trichrome staining (Fig. 5*A*) and hydroxyproline measurement by HPLC (Fig. 5*B*). This increase in collagen content in CXCR5^−/−^ mice was accompanied by a significant increase in total matrix metalloproteinase (MMP) activity (Fig. 5*C*) and gelatinolytic activity (Fig. 5*D, E*). This combination of increased collagen content and increased MMP activity suggest enhanced matrix remodeling in CXCR5 deficient mice following AB.

### Microarray analysis identified altered expression of genes encoding non-collagen ECM proteins

In addition to collagen, the quantity and quality of other ECM constituents also importantly influence cardiac function [31]. We therefore performed microarray analysis (Affymetrix) of the myocardium from WT and CXCR5^−/−^ mice 3 weeks after AB. The seeded Bayesian network method [32] was used to explore interactions between differentially expressed genes. This analysis identified a cluster of genes encoding ECM proteins. Interestingly, this cluster contained fibromodulin which belongs to a family of small leucine-rich repeat proteoglycans (SLRPs), which are known to influence ECM assembly [33]. Microarray data are accessible through GEO Series accession number GSE22295 (http://www.ncbi.nlm.nih.gov/geo/query/acc.cgi?acc=GSE22295).

### Protein levels of SLRPs following AB in CXCR5^−/−^ and WT mice

**Figure 6:**
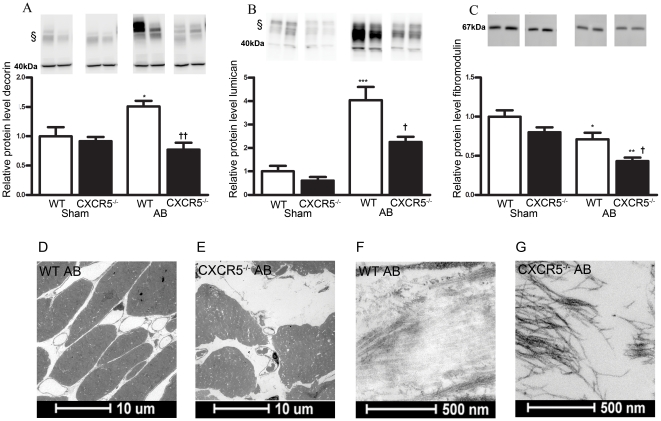
Protein levels of small leucine-rich repeat proteoglycans (SLRPs) and transmission electron microscopic analysis in wild type (WT) and CXCR5^−/−^ mice following aortic banding (AB). Protein levels of (**A**) decorin and (**B**) lumican in (WT) Sham (n = 7), CXCR5^−/−^ Sham (n = 5), WT AB (n = 6), and CXCR5^−/−^ AB (n = 5), and (**C**) fibromodulin in WT Sham (n = 13), CXCR5^−/−^ Sham (n = 12), WT AB (n = 12), and CXCR5^−/−^ AB (n = 11) groups as assessed by western blotting. The upper panels show representative blots from two mice in each group. § Glycosylated forms of decorin and lumican, respectively. The results are mean ± SEM. *p<0.05, **p<0.001 and ***p<0.001 *vs.* Sham groups; †p<0.05 and ††p<0.01 *vs.* WT AB group. The lower panels show representative transmission electron micrographs of the LV free wall at ×440 and ×23000 magnification in WT (**D** and **F**) and CXCR5^−/−^ (**E** and **G**) AB mice.

To further examine the regulation of SLRPs following AB, we measured protein levels of fibromodulin and other members in the SLRP family, including decorin, lumican and biglycan. With the exception of biglycan, all of these SLRPs were differently regulated in CXCR5 ^−/−^ mice compared to WT mice following AB (Fig. 6). While decorin and lumican markedly increased during AB in WT mice, this was not observed in CXCR5^−/−^ mice (Fig. 6*A, B*). Moreover, while fibromodulin decreased following AB in WT and CXCR5 ^−/−^ mice, the decrease was significantly more pronounced in the CXCR5^−/−^ mice (Fig. 6*C*).

### Electron microscopic analysis revealed loosely packed ECM in CXCR5^−/−^ mice following AB

SLRPs are capable of binding to different types of collagen [34], [35], thereby regulating fibril assembly and organization, degradation, and quantitative and functional aspects of the collagen network [33]. To further elucidate the effect of decreased levels of SLRPs during pressure overload, LV tissue sections were examined by electron microscopic analysis in WT and CXCR5-deficient mice. As shown in Fig. 6*D* and *E* *,* a considerable increase in the extracellular space was observed in LVs from CXCR5^−/−^ as compared to WT mice. In addition, banded WT mice exhibited densely packed collagen fibers of variable thickness and orientation as well as proteoglycan particles associated with fine filaments (Fig. 6*F*). In contrast, the ECM in CXCR5^−/−^ mice exhibited large areas with a coarse network of individual collagen fibrils, and finer networks of proteoglycans with associated filaments (Fig. 6*G*), suggesting deranged ECM.

### No difference in apoptosis and leukocyte infiltration between CXCR5^−/−^ and WT mice after pressure overload

CXCL13 has been suggested to exhibit anti-apoptotic properties [36], [37]. However, analysis of cardiac apoptosis by *in situ* TUNEL staining revealed that, after either sham-operation or AB, the number of apoptotic cells was similar in WT and CXCR5-deficient mice (Fig. S2). Moreover, although CXCL13 is known to influence lymphocyte trafficking [38], [39], we found no significant difference in infiltration of CD3^+^ or CD45^+^ cells between CXCR5^−/−^ and WT mice after either sham operation or AB (Fig. S3 *A* and *B*).

### CXCL13 stimulates expression of SLRPs

**Figure 7:**
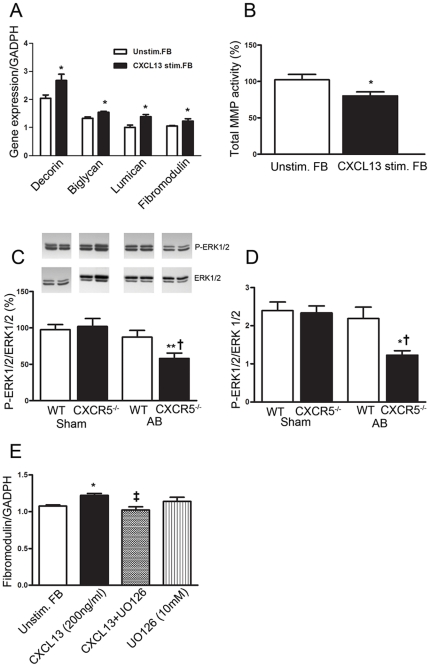
Effects of CXCL13 on gene expression of small leucine-rich repeat proteoglycans (SLRPs), total MMP activity and the intracellular signalling pathway ERK 1/2 in neonatale rat fibroblasts (FB). The effect of CXCL13 (200 ng/ml) stimulation on gene expression of (**A**) decorin, biglycan, lumican and fibromodulin (n = 4) in myocardial FB after 20 hours of stimulation in relation to GAPDH and (**B**) total MMP activity in cell-free supernatant (n = 7) after 20 hours of stimulation. The ratio of phosphorylated ERK 1/2 (p-ERK 1/2) to total ERK 1/2 assessed by (**C**) western blotting in WT Sham (n = 13), CXCR5^−/−^ Sham (n = 12), WT AB (n = 11), and CXCR5^−/−^ AB (n = 10) groups, and by (**D**) BioPlex in WT Sham (n = 6), CXCR5^−/−^ Sham (n = 5), WT AB (n = 7), and CXCR5^−/−^ AB (n = 6) groups. (**E**) The effect of blocking ERK 1/2 activation with UO126 (10 µM) on the CXCL13-mediated induction of fibromodulin gene expression in myocardial FB (n = 4). The results are mean ± SEM. *p<0.05 and **p<0.01 *vs.* unstimulated FB or CXCR5^−/−^ Sham; †p<0.05 *vs*. WT AB group, ‡p<0.05 vs. CXCL13 stimulated FB without inhibitor (UO126).

The changes in ECM in CXCR5^−/−^ mice following AB, consisting of enhanced MMP activity and decreased expression of several SLRPs, could potentially reflect direct effects of CXCL13 on myocardial fibroblasts. In fact, both CXCL13 and CXCR5 were strongly expressed within myocardial fibroblasts, and CXCR5 showed enhanced myocardial expression during AB in WT mice (Fig. 1). In addition, fibroblasts are important producers of ECM proteins, including SLRPs. Stimulation of cardiac neonatal rat fibroblasts with CXCL13 did indeed enhance the expression of fibromodulin, biglycan and lumican, and in particular of decorin, and at the same time down-regulated total MMP activity (Fig. 7*A, B*).

### CXCL13-CXCR5 mediate their effects on matrix modulation via ERK1/2 signaling

We next examined alterations in the ERK1/2 pathway in CXCR5^−/−^ following AB, as this pathway is of importance in cardiac remodeling and since CXCL13 signaling through CXCR5 is known to activate the MAPK pathway via ERK 1/2 [40], [41]. As shown in Fig. 7*C and D*, LV from CXCR5^−/−^ mice showed decreased levels of phosphorylated ERK1/2 as compared with WT mice. In cardiac fibroblasts, blocking the ERK1/2 pathway by UO126, a highly selective inhibitor of MEK 1 and 2, significantly attenuated up-regulation of fibromodulin following CXCL13 treatment (Fig. 7*E*).

### Expression of CXCR5 and SLRPs in patients with HF

**Figure 8:**
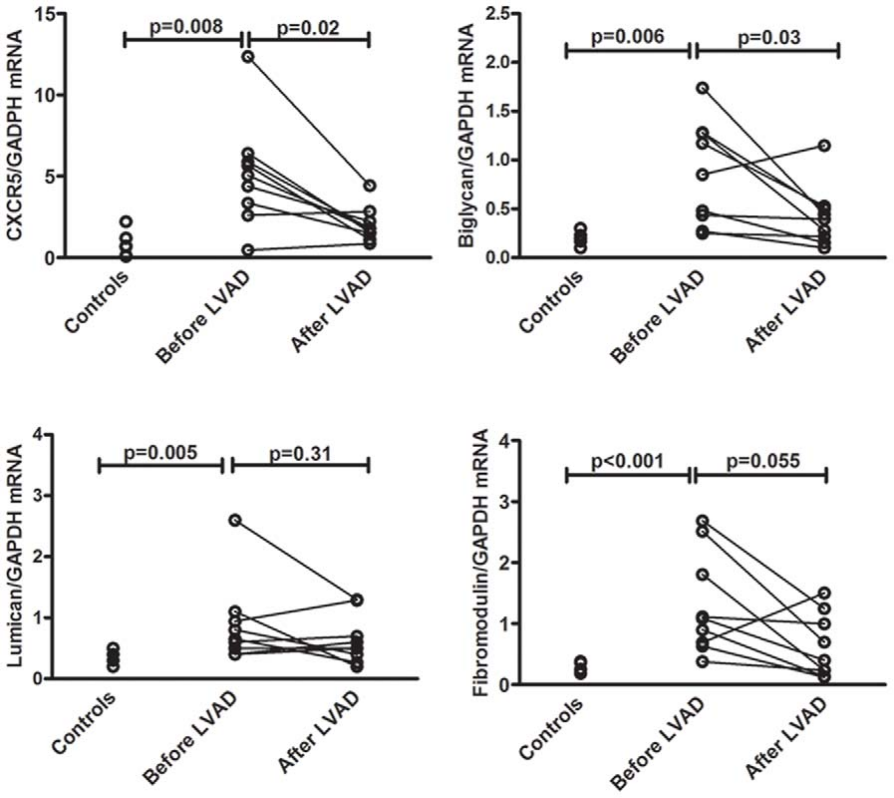
Gene expression of CXCR5, biglycan, lumican and fibromodulin in patients with heart failure (HF) (n = 9) and controls (n = 5) as assesssed by quantitaive real time PCR. In the HF patients, the mRNA levels were measured before and after treatment with continuous-flow left ventricular assist device (LVAD).

Assessments of CXCR5 mRNA levels in myocardial tissue from 9 HF patients (all with advanced HF, NYHA class IV) and 5 controls (non-failing hearts) showed that HF patients had markedly enhanced gene expression of CXCR5 (84% increase, p<0.005). As shown in Fig. 7, the 9 HF patients also had significantly enhanced gene expression of biglycan, lumican and fibromodulin. When the HF patients were treated with continuous-flow LV assist device (LVAD) for 8±1.7 months, improvement in hemodynamic parameters (LV end diastolic volume decreased from 294.9 mL to 237.8 mL, LV diastolic diameter from 7.6 cm to 6.8 cm, and LV end systolic volume from 244.8 mL to 183.4 mL, reflected in an increase in LVEF from 18.2 to 29.6%, p<0.05 for all) was accompanied by a marked decrease in mRNA levels of CXCR5 as well as biglycan and fibromodulin, although the decrease in fibromodulin did not reach statistical significance (Fig. 8).

## Discussion

Despite observations of enhanced levels of chemokines and their corresponding receptors in human HF [5], [42], [43], the role of chemokines in maintaining cardiac structure and function has never been established. The present work clearly demonstrates that the chemokine CXCL13 and its receptor, CXCR5, are critically involved in cardiac remodeling. The key results of this study were increased mortality and severe LV dilatation in CXCR5-deficient mice in response to pressure overload, potentially resulting from impaired quality of ECM. These ECM alterations derived, at least partly from decreased SLRP levels and enhanced MMP activity within the myocardium. Our *in vitro* findings showed that CXCL13 can promote SLRP expression and attenuate MMP activity in myocardial fibroblasts. Therefore, the opposite pattern seen in CXCR5-deficient mice could reflect the inability of their myocardial fibroblasts to respond to CXCL13. These data indicate that the CXCL13/CXCR5 interaction is involved in myocardial remodeling following pressure overload, possibly by regulating proteoglycans crucial for the quality of myocardial ECM. Our findings of a strong expression of CXCL13 and CXCR5 in fibroblasts within murine hearts and enhanced myocardial expression of CXCR5 during AB further support such a notion.

The possible role of chemokines in the pathogenesis of HF has, at least in part, been attributed to the ability of these molecules to promote leukocyte infiltration in failing myocardium. However, in the present study we did not detect altered infiltration of CD3^+^ and CD45^+^ cells in CXCR5^−/−^ mice compared to the WT mice following AB. Previously, various chemokines (e.g., CXCL16, MCP-1 and CX3CL1) have been shown to promote direct effects on myocardial fibroblasts and cardiomyocytes *in vitro* [5], [43]–. Also, the lack of CXCR4 has been associated with severe myocardial developmental defects (i.e., ventricular septum defects) [46]. In contrast to CXCR4, CXCR5 deficient mice are viable and display normal morphology and function of the adult heart. However, our present data showing a markedly dilating myocardial phenotype in CXCR5^−/−^ mice exposed to pressure overload, without any significant changes in myocardial leukocyte infiltration, may suggest a direct involvement of CXCL13/CXCR5 activation in myocardial remodeling. The ability of CXCL13 to attenuate MMP activity and increase SLRP expression in myocardial fibroblasts as well as the up-regulation of CXCR5 during AB in WT mice further supports such a notion.

Collagen synthesis, fibrillogenesis, and matrix degradation must be finely tuned, as an imbalance in these processes might result in cardiac dilatation, cardiac hypertrophy and fibrosis. Although we observed increased total collagen content in CXCR5^−/−^ mice, these mice were also characterized by massively disturbed structural frameworks after AB compared to WT. Our molecular analysis suggested that this distorted ECM structure derives from a failure in the regulation of SLRPs in pressure overloaded CXCR5-deficient mice. SLRPs are known to bind to collagens, and in so doing, regulate the self-assembly process of pro-collagen into fibrils [33]–[35]. This assembly is necessary for covalent cross-linking which is required for reinforcement of the collagen fibrils. SLRPs have been shown to be up-regulated in the infarcted area in rats and mice following myocardial infarction (MI) [47], [48]. Studies in SLRP deficient mice have shown abnormal fibril organization and loose fibril packing in the MI scar [49], [50]. In the current study we show an attenuated up-regulation (i.e., decorin and lumican) and a more pronounced decrease (i.e., fibromodulin) in SLRP expression following AB in CXCR5 deficient mice as compared with WT mice. Interestingly, enhanced MMP activity has been found to impair SLRP function [44], and we suggest that the combination of decreased SLRP expression and increased MMP activity could be of major importance for the premature LV dilatation and HF in CXCR5^−/−^ mice following AB.

Our *in vitro* data suggest that CXCL13 via activation of CXCR5 on myocardial fibroblasts induces the expression of SLRPs through the ERK1/2 pathway. This mechanism appears to be absent in fibroblasts from CXCR5^−/−^ mice. ERK is one of the key protein kinases that regulate growth and proliferation of cardiac fibroblasts [40]. In line with a crucial role of CXCL13/CXCR5-mediated ERK1/2 activation for cardiac remodeling in response to pressure overload, ERK1/2 phosphorylation was substantially lower in CXCR5^−/−^ mice compared to WT after AB. These findings further support previous reports of a central role of the ERK pathway for myocardial remodeling [51], [52]. In this regard, the present study adds a novel component to this pathway by linking CXCR5-mediated effects to SLRPs and subsequent ECM remodeling.

Our studies in patients with advanced HF suggest that our findings in experimental HF may have relevance to clinical HF. We showed enhanced myocardial expression of CXCR5 and certain SLRPs (i.e., biglycan, lumican and fibromodulin) in the failing myocardium, and notably, biglycan and lumican were down-regulated following the clinical and hemodynamic improvement during treatment with LV assist device. Based on our experimental data, it is tempting to speculate that CXCR5 activation promotes protective responses in ECM in failing myocardium involving enhanced expression of SLRPs. As myocardial function improves, this response is attenuated. At present, however, the stimuli for enhanced myocardial CXCR5 expression in HF and the interpretation of our human data will require further investigation.

In conclusion, we have found that CXCR5 plays an important role in cardiac remodeling during pressure-overload. Loss of CXCR5 in this situation adversely affects matrix remodeling and causes LV dilatation, possibly through altered regulation of proteoglycans crucial for the quality of myocardial ECM (i.e., SLRPs) as well as through enhanced MMP activity. These changes could, at least in part, be attributed to loss of CXCL13 mediated effects on myocardial fibroblasts in CXCR5 deficient mice. The identification of molecular and structural changes causing LV dilatation during pressure overload is of major importance for the development of new treatment strategies in HF related to hypertension and aortic stenosis. Future studies should examine how the CXCL13/CXCR5 dyad could be utilized therapeutically in clinical HF.

## Materials and Methods

### Ethics

All animal experiments were approved by the Norwegian Animal Research Committee (ID 1902) and conform to the Guide for the Care and Use of Laboratory Animals published by the US National Institutes of Health (NIH Publication No. 85–23, revised 1996). The part of the study that involved humans was approved by the local ethics committee (REK Helse Sør-Øst) and conducted according to the ethical guidelines outlined in the Declaration of Helsinki for use of human tissue and subjects. Informed written consent was obtained from all subjects. The authors had full access to the data and take responsibility for its integrity. All authors have read and agree to the manuscript as written.

### Data analysis

All data are expressed as group means ± SEM unless indicated otherwise. For comparisons of 2 groups, the Mann-Whitney *U* test was employed. The Wilcoxon test was employed when analysing the effect of LVAD treatment. Differences between the WT Sham, WT AB, CXCR5^−/−^ Sham, and CXCR5^−/−^ AB groups were determined by one-way ANOVA with post-hoc Tukey test. Differences in survival between wild type and CXCR5^−/−^ mice in Fig. 3 were compared using Kaplan- Meier survival curves and tested with the log-rank test. All tests were employed using a 5% significance level.

### Animals and AB protocol

Mice were housed in M2 or M3 cages with Bee Kay bedding (Scanbur BK, Nittedal, Norway) in 55% humidity on a 12 h light/dark cycle. Food pellets (RM1, 801151, Scanbur BK) and water were freely available. All mice utilized in this study were male and had a weight 20–30 g. WT C57BL/6 mice were obtained from Taconic (Skensved, Denmark). The generation of CXCR5^−/−^ mice (C57BL/6 background, now accessible at the Jackson Laboratory, stock number 006659, strain name B6.129S2 (Cg)-*Cxcr5^tm1Lipp^*/J has been described previously [14]. Briefly, gene targeting was performed in 129S2/SvPas-derived D3 embryonic stem cells, replacing the coding region of the CXCR5 gene with a neomycin resistance gene. Mutant mice were backcrossed to C57BL/6 mice for 8 generations. AB was induced in C57BL/6 and CXCR5^−/−^ mice as previously described [53]. Briefly, after being anesthetized with 5% isoflurane and ∼98% oxygen in a gas chamber, the animals where endotracheal intubated and the cannula was connected to a volume cycled rodent ventilator (Harvard Apparatus) on supplement of a mixture of ∼1.75% isoflurane and ∼98% oxygen. A thoracotomy was performed in the second intercostal space on the left side, and the aortic constriction was created by placing a ligature securely around the ascending aorta and a 26-gauge needle and then removing the needle. Sham operated animals underwent the same procedure except for aortic constriction. The animals were extubated after getting a dose of analgesic (buprenorphine, 0.1 mg/kg) subcutaneously and allowed to recover. Doppler echocardiography was performed 21 days after primary surgery under general anaesthesia with isoflurane as described above. This time point was selected since previous studies in our laboratory have shown marked hypertrophy in WT mice at this stage. AB mice in both groups with a flow velocity across the aortic banding site greater than 3.5 m/s were included in the study. The same mice anesthetized with isoflurane were euthanized by dislocation of the neck, and the hearts and lungs were removed and blotted dry. The right ventricle and atria were removed. The LV, right ventricular free wall and lungs were weighed and normalized to tibial length.

### Doppler Echocardiography

Mice were examined while sedated in the supine position with the chest closed, as previously described [54]. Echocardiography was performed using a i13L 13 MHz linear array transducer (GE Healthcare Technologies, Oslo, Norway) and data were analyzed with EchoPac PC software (GE Healthcare Technologies, Oslo, Norway) as described [55]. The data were recorded and analyzed by a cardiologist (IS), blinded for the genotype.

### Isolation of adult myocardial murine cardiomyocytes, fibroblasts and endothelial cells

Mouse cardiomyocytes were isolated as previously described [56]. Endothelial cells were enriched from the non-cardiomyocyte fraction by labeling the cells with a rat anti-mouse CD31 antibody (eBioscience, San Diego, CA) and subsequent extraction using an anti-rat secondary antibody coupled to magnetic beads (Miltenyi Biotec, Auburn, CA) and further column purifications according to the protocols provided by the manufacturer (http://www.miltenyibiotec.com). The cell fraction remaining after extraction of cardiomyocytes and endothelial cells contains predominantly fibroblasts.

### RNA isolation

Total RNA was isolated from the LV in WT and CXCR5^−/−^ mice (SV total RNA isolation system, Promega, Inc., Madison, WI), mouse and neonatal rat cardiomyocytes and fibroblasts, and mouse endothelial cells (RNeasy mini kit, Qiagen, Valencia, CA) as previously described [5].

### Quantitative real-time PCR (qRT-PCR)

Reverse transcription reactions were performed with iScript Select cDNA Synthesis Kit (Bio-Rad Laboratories, Inc., Hercules, CA). Pre-designed TaqMan assays (Applied Biosystems, Foster City, CA) were used to determine gene expression of CXCL13(Mm00444534_m1), CXCR5 (Mm00432086_m1), ANP (Mm01255748_g1), BNP (Mm00435304_g1), β-MHC (Mm01319006_g1), biglycan (Rn00567229_m1), fibromodlin (Rn00589918_m1), lumican (Rn00579127_m1) and decorin (Rn01503161_m1). The results were detected on an ABI PRISM 7900 Sequence Detection System (Applied Biosystems) as described previously [5]. In the human studies, quantification of gene expression was performed using the ABI Prism 7500 (Applied Biosystems), Power SYBR Green Master Mix (Applied Biosystems), and sequence-specific PCR primers were designed using the Primer Express software, version 3.0 (Applied Biosystems). List of the real-time PCR assays used in the human study is shown in Table S2.

### Perfusion fixation and histology

After the hearts were excised and rinsed in cold NaCl solution, the aorta was cannulated, and the hearts were mounted on a Langendorff setup, and retrogradely perfused with warm (37°C) oxygenated Thyrodes solution (5 mM Hepes, pH 7.4, 140 mM NaCl, 5.4 mM KCl, 0.4 MgH_2_PO_4_, 0.5 MgCL_2_) with 1.8 mM Ca^2+^. Hearts were stopped in diastole by aortic perfusion of Thyrodes solution with high KCl (10.8 mM) for 3 min and fixated for 10 min by perfusion of 4% phosphate-buffered formalin. After the cannulas were removed, fixation by immersion continued for 2 h. Each heart was transected at the midventricular level, and both halves were routinely processed and embedded in paraffin. Paired 3.5 µm sections were prepared, mounted on glass slides, and stained with hematoxylin and eosin and Masson tricrome stain.

### Hydroxyproline analysis

Quantitative analysis of tissue levels of hydroxyproline was performed by HPLC using the AccQ-Fluor reagent kit (Waters Corporation, Milford, MA) essentially as previously described [57]. Briefly, cardiac tissue samples (5 mg dry weight) were hydrolyzed in 6 M HCl for 16 h at 110°C and subsequently dried under vacuum and redissolved in the AccQ-Fluor borate buffer. Derivatization was initiated by addition of the AccQ-Fluor reagent at 55°C and terminated after 10 min. The samples were finally subjected to HPLC-chromatography using a 20×3.9 mm Sentry Guard column (Nova-Pak C_18_ bonded silica) connected to a 150×3.9 mm AccQ-Tag reversed-phase column (both from Waters) according to the manufacturer's instructions. Derivatized hydroxyproline was detected by fluorescence signal following excitation at 250 nm and recording of emission at 395 nm. Elution of hydroxyproline from myocardial tissue samples was verified and quantified by co-elution with known amounts of derivatized hydroxyproline standards (Fluka, Buchs SG, Switzerland). The relation of myocardial hydroxyproline contents to myocardial collagen has previously been reported [58].

### Measurements of total MMP activity and gelatinolytic activity

Total MMP activity in the LV was measured by a fluorogenic peptide substrate (R&D Systems) used to assess broad-range MMP activity (MMP-1, -2, -7, -8, -9, -12 and -13 can cleave the peptide). Gelatinolytic activity was assessed by gelatine zymography. Briefly, the MMP substrate was diluted in TCN buffer (50 mM Tris HCl, 150 mM NaCl, 10 mM CaCl_2_; pH 7.5) and added to the supernatants before incubation at 37°C. After 120 min the total MMP activity was determined on a fluorimeter (FLX 800 Microplate Fluorescence Reader, Bio-Tek Instruments, Winooski, VT).

### Gene expression profiling and microarray data analysis

Total RNA was isolated from the LV in Sham (n = 3) and AB (n = 4) WT and CXCR5^-/-^ mice as described previously [5]. Preparation of cRNA and the subsequent steps leading to hybridization of Affymetrix GeneChip® mouse ST 1.0 arrays (Affymetrix, Santa Clara, CA), washing, and scanning were performed according to standard protocols (Affymetrix). Microarray preprocessing was done using robust multi-array average [59]. Differentially expressed genes were found using significance analysis of microarrays [60]. The seeded Bayesian network method [32] was used to explore interactions between differentially expressed genes. This method finds interactions in the expression data using literature co-citations, databases of protein-protein interactions, as well as co-regulations in the expression data. We constructed two networks; one for the wild type situation, using the 60 most differentially expressed genes between AB WT and SHAM WT, and similarly one for the KO situation, using the 60 most differentially expressed between CXCR5^−/−^ AB and Sham AB. The set of 60 genes were based on a ranking according to fold change (AB *vs.* Sham) using a subset of the genes for which false discovery rate was less than 0.05. All data is MIAME compliant and the following link has been created to allow review of the data in Gene Expression Omnibus (record GSE22295): http://www.ncbi.nlm.nih.gov/geo/query/acc.cgi?token=xdivnmkmeyeiifg&acc=GSE22295

### Western blotting

Western blotting was performed as previously described [61] with minor modifications. Snap frozen left ventricles from WT and CXCR5^−/−^ mice were homogenized in cell lysis buffer and equal amounts of protein being separated from each sample by SDS-PAGE (10%) before transferred to polyvinylidene fluoride (PVDF) membranes. Non-specific bindings to the membrane was blocked with 5% BSA for 1 h at room temperature, followed by incubation with anti-fibromodulin (SC-33772; Santa Cruz Biotechnology, Inc., Santa Cruz, CA), anti-decorin (AF1060; R&D Systems, Minneapolis, MN), anti-biglycan (AF2667; R&D) or anti-lumican (AF2745; R&D) overnight at 4°C. The membranes were washed in TBS-T and followed by species-specific horseradish peroxidase-coupled secondary antibodies in 5% BSA added for 1 h. After washing, the immune complexes were visualized by ECL (GE Healthcare, Buckinghamshire, UK) and the membranes were exposed to x-ray film (HyperfilmTM ECL, GE healthcare) and developed. Immunoblots were stripped and re-probed with anti-GAPDH (C20357; R&D) for normalization. Filters with LV lysate were also probed with antibody against P-ERK1/2 (Phospho-p44/42 MAP kinase (Thr 202/Tyr 204), Cell Signaling Technology, Danvers, MA) or p44/42 MAP kinase (Cell Signaling Technology) followed by species-specific horseradish peroxidase-coupled secondary antibodies (Cell Signaling). The immune complexes were visualized with the use of Supersignal West Pico (Pierce, Rockford, IL) and exposed films were detected by using Kodak 440 CF imaging station (Boston, MA). The software Total Laboratory v.1*10 (Phoretix, Newcastle, UK) was used for quantification.

### Transmission electron microscopy

For transmission electron microscopy, hearts (n = 2) from each group were perfused with 2.0% glutaraldehyde buffered in 0.2 M cacodylate at pH 7.4 for 15 min. Small blocks (about 3 mm^3^ in size) of the LV and septum were taken. The tissues were fixated in cacodylate buffer for 2 h and washed in the same buffer 3 times. The blocks of tissue were then transferred to a 1% OsO_4_ solution for 10 min on ice. After washing in cacodylate, dehydration was carried out rapidly in graded ethanol series, followed by embedding in Epon. Sections were cut at a thickness of 60–100 nm and collected on 200 mesh grids, and stained with uranyl acetat for 7 minutes and lead for 3 minutes. The sections were examined and photographed in a Tecnai G2 spirit BioTWIN 120 kV, LaB6, Transmission Electron Microscope with 4k Eagle camera from FEI Company. We obtained micrographs of the LV septum and the free wall at different magnifications.

### TUNEL Staining

TUNEL staining was performed on paraffin-embedded sections using the *In Situ* Cell Death Detection kit (Roche Diagnostics) as described [62]. Briefly, paraffin-embedded (6 µm) sections of mouse hearts were deparaffinized in xylene, rehydrated, and treated with 0.5% Triton X-100 in 0.1% Na-citrate for 30 min. After several washes with PBS, the sections were permeabilized with proteinase K (20 µg/ml in TE, pH 8.0) for 30 min at 37°C. Subsequently, the sections were rinsed with PBS, and the area around the sample was dried. TUNEL reaction mixture (50 µl) containing terminal deoxynucleotidyl transferase was applied and tissue sections were incubated in a dark, humidified chamber for 1 h at 37°C. After several washes with PBS the tissue sections were analyzed with a fluorescence microscope (515–565 nm). A quantitative analysis (number of apoptotic cells/total number of cells counted) was performed by counting cells in a randomly selected area of each tissue sample.

### Immunohistochemistry

Paired 3.5 µm sections were immunostained using affinity-purified rabbit polyclonal CD3 antibody (Abcam, Cambridge, U.K.), dilution 1∶400, and anti-mouse/human CD45R (eBioscience, San Diego, CA), dilution 1∶4500. The immune reaction was visualized using horseradish peroxidase in a Dako Autostainer plus (Dako, Glostrup, Denmark). We obtained 32 digital images of evenly distributed microscopic high power fields (×400) from the left ventricular free wall and ventricular septum of six heart sections in each group. The sections were from no less than three hearts in each group. The 32 images from each heart were studied by two investigators (AW and HMR), blinded for mouse identity, counting the total number of CD3 and CD45R positive lymphocytes.

### Isolation and stimulation of neonatal myocardial rat fibroblasts

Primary neonatal fibroblasts were isolated from 1–3 day old Wistar rats (Taconic, Skensved, Denmark). Briefly, fibroblasts were separated by Percoll density gradient and transferred to plating medium and maintained in culture for up to 96 hours. The fibroblasts were stimulated with human recombinant CXCL13, 200 ng/ml (R&D Systems, Minneapolis, MN), with or without the ERK1/2 inhibitor (10 µM final concentration UO126 (MEK inhibitor, Promega, WI)), for 3 and 20 hours before storing cell pellet (mRNA analyses) and cell-free supernatant (MMP activity) at −80°C until further analyses. Un-stimulated (control) cells were also given vehicle. The toxicity in cell cultures was examined routinely for lactate dehydrogenase leakage using a cytotoxicity detection kit (Roche Applied Science, Mannheim, Germany).

### P-ERK1/2 (Phospho-p44/42 MAP kinase) and ERK1/2 (p44/42 MAP kinase) detection

Phospho-p44/42 MAP kinase and p44/42 MAP kinase levels in left ventricle lysate were measured by multiplex suspension array technology using the BioPlex (Bio-Rad, Hercules, CA). Phospho-p44/42 MAP kinase and p44/42 MAP kinase multiplexable beads were purchased from R&D Systems. The quantification was accomplished by using the BioPlex Manager Software (Bio-Rad).

### Tissue sampling from human myocardium

In nine patients with advanced HF (NYHA class IV; 8 male, 1 female; age 29±5 years), LV tissue was available at the time of implantation and at the time of removal (heart transplantation) of a continuous-flow LV assist device (LVAD; EntrAssist, Ventracor Ltd, Chatswood, Australia). Average time on LVAD was 8±1.7 months. Control (non-failing) human LV tissue was obtained from subjects whose hearts were rejected as cardiac donors for surgical reasons (n = 5). The cause of death of donors was cerebrovascular accident, and none had a history of heart disease. Myocardium from these subjects was kept on ice for 1 to 4 hours before tissue sampling was conducted. In both failing and non-failing myocardium, LV tissue samples were snap-frozen in liquid nitrogen, and stored at −80°C until use. None of patients (failing and non-failing myocardium) had significant concomitant disease such as infection, malignancy, or autoimmune disorder.

## Supporting Information

Figure S1 **Altered expression of markers of cardiac wall stress and remodeling.** Relative gene expression of (**A**) atrial natriuretic peptide (ANP), (**B**) brain natriuretic peptide (BNP) and (**C**) β-myosin heavy chain (MHC) in wild type (WT) Sham (n = 6), CXCR5^-/-^ Sham (n = 6), WT aorta banded (AB) (n = 6), and CXCR5^-/-^ AB (n = 6) groups. The results are mean ± SEM. *p<0.05 and ***p<0.001 *vs*. Sham groups; †p<0.05 and †††p <0.001 *vs*. WT AB group.
(TIF)

Figure S2 **Fluorescent micrographs of sections of left ventricular myocardium from wild type (WT) and CXCR5^-/-^ mice.** The arrows indicate TUNEL-positive myocyte nucleus.
(TIF)

Figure S3 **CD45 and CD3 positive lymphocytes in the left ventricular myocardium from wild type (WT) and CXCR5^-/-^ mice.** Total number of CD45R (**A**) and CD3 (**B**) postive lymphocytes was not significantly different between CXCR5^-/-^ and WT mice after sham operation or AB. (n = 6 heart sections in all groups). Cells counted from 32 digital, evenly distributed images (x400) from each heart.The results are mean ± SEM.
(TIF)

Table S1 **Weights and echocardiographic measurements.** Values are means ± SE. BW, body weight; TL, tibia length; LVW, left ventricular weight; LW, lung weight; IVSd and IVSs, interventricular septum thickness in diastole and in systole, respectively; LVD_d_ and LVD_s_, left ventricular diameter in diastole and in systole, respectively; FS, fractional shortening in LVD; LVPWd and LVPWs, posterior wall thickness in diastole and in systole, respectively; LAD, left atrial diameter; HR, heart rate; AVmax, peak aortic stenosis flow velocity; TVs, peak tissue velocity in systole; TVd, peak tissue velocity in diastole. LVW/TL and LW/TL are mg/mm. * p<0.05, ** p<0.01, *** p<0.001 vs. Non-operated and Sham groups. †p<0.05, ††, p <0.01, †††p<0.001 vs. WT AB group. ‡p<0.05 vs CXCR5^-/-^ Non-operated group. The results are mean ± SEM.
(DOC)

Table S2 **Characteristics of the real-time PCR assays used in the human study.** The table shows the sequence of primers used in the real-time PCR assays. (+), forward primers; (–), reverse primers; Acc.nr, GenBank accession number; GAPDH, glyceraldehyde 3-phosphate dehydrogenase.
(DOC)

## References

[1] Aukrust P, Ueland T, Lien E, Bendtzen K, Müller F (1999). Cytokine network in congestive heart failure secondary to
ischemic or idiopathic dilated cardiomyopathy.. Am J Cardiol.

[2] Torre-Amione G, Kapadia S, Lee J, Durand JB, Bies RD (1996). Tumor necrosis factor-alpha and tumor necrosis factor receptors
in the failing human heart.. Circulation.

[3] Damås JK, Gullestad L, Aass H, Simonsen S, Fjeld JG (2001). Enhanced gene expression of chemokines and their corresponding
receptors in mononuclear blood cells in chronic heart
failure—modulatory effect of intravenous
immunoglobulin.. J Am Coll Cardiol.

[4] Woldbæk PR, Sande JB, Strømme TA, Lunde PK, Djurovic S (2005). Daily administration of interleukin-18 causes myocardial
dysfunction in healthy mice.. Am J Physiol Heart Circ Physiol.

[5] Husberg C, Nygard S, Finsen AV, Damås JK, Frigessi A (2008). Cytokine expression profiling of the myocardium reveals a role
for CX3CL1 (fractalkine) in heart failure.. J Mol Cell Cardiol.

[6] Mann DL (2001). Recent insights into the role of tumor necrosis factor in the
failing heart.. Heart Fail Rev.

[7] Matsumori A, Sasayama S (2001). The role of inflammatory mediators in the failing heart:
immunomodulation of cytokines in experimental models of heart
failure.. Heart Fail Rev.

[8] Dibbs Z, Kurrelmeyer K, Kalra D, Seta Y, Wang F (1999). Cytokines in heart failure: pathogenetic mechanisms and potential
treatment.. Proc Assoc Am Physicians.

[9] Förster R, Emrich T, Kremmer E, Lipp M (1994). Expression of the G-protein—coupled receptor BLR1 defines
mature, recirculating B cells and a subset of T-helper memory
cells.. Blood.

[10] Förster R, Davalos-Misslitz AC, Rot A (2008). CCR7 and its ligands: balancing immunity and
tolerance.. Nat Rev Immunol.

[11] Müller G, Höpken UE, Lipp M (2003). The impact of CCR7 and CXCR5 on lymphoid organ development and
systemic immunity.. Immunol Rev.

[12] Ohl L, Henning G, Krautwald S, Lipp M, Hardtke S (2003). Cooperating mechanisms of CXCR5 and CCR7 in development and
organization of secondary lymphoid organs.. J Exp Med.

[13] Ebert LM, Schaerli P, Moser B (2005). Chemokine-mediated control of T cell traffic in lymphoid and
peripheral tissues.. Mol Immunol.

[14] Förster R, Mattis AE, Kremmer E, Wolf E, Brem G (1996). A putative chemokine receptor, BLR1, directs B cell migration to
defined lymphoid organs and specific anatomic compartments of the
spleen.. Cell.

[15] Cyster JG, Ansel KM, Reif K, Ekland EH, Hyman PL (2000). Follicular stromal cells and lymphocyte homing to
follicles.. Immunol Rev.

[16] Gunn MD, Ngo VN, Ansel KM, Ekland EH, Cyster JG (1998). A B-cell-homing chemokine made in lymphoid follicles activates
Burkitt's lymphoma receptor-1.. Nature.

[17] Weyand CM, Goronzy JJ (2003). Ectopic germinal center formation in rheumatoid
synovitis.. Ann N Y Acad Sci.

[18] Ansel KM, Heyzer-Williams LJ, Ngo VN, Heyzer-Williams MG, Cyster JG (1999). In vivo-activated CD4 T cells upregulate CXC chemokine receptor 5
and reprogram their response to lymphoid chemokines.. J Exp Med.

[19] Haynes NM, Allen CD, Lesley R, Ansel KM, Killeen N (2007). Role of CXCR5 and CCR7 in follicular Th cell positioning and
appearance of a programmed cell death gene-1high germinal center-associated
subpopulation.. J Immunol.

[20] Schmutz C, Hulme A, Burman A, Salmon M, Ashton B (2005). Chemokine receptors in the rheumatoid synovium: upregulation of
CXCR5.. Arthritis Res Ther.

[21] Qiuping Z, Jie X, Youxin J, Qun W, Wei J (2005). Selectively frequent expression of CXCR5 enhances resistance to
apoptosis in CD8(+)CD34(+) T cells from patients with
T-cell-lineage acute lymphocytic leukemia.. Oncogene.

[22] Shi K, Hayashida K, Kaneko M, Hashimoto J, Tomita T (2001). Lymphoid chemokine B cell-attracting chemokine-1 (CXCL13) is
expressed in germinal center of ectopic lymphoid follicles within the
synovium of chronic arthritis patients.. J Immunol.

[23] Amft N, Curnow SJ, Scheel-Toellner D, Devadas A, Oates J (2001). Ectopic expression of the B cell-attracting chemokine BCA-1
(CXCL13) on endothelial cells and within lymphoid follicles contributes to
the establishment of germinal center-like structures in Sjogren's
syndrome.. Arthritis Rheum.

[24] Salomonsson S, Larsson P, Tengner P, Mellquist E, Hjelmström P (2002). Expression of the B cell-attracting chemokine CXCL13 in the
target organ and autoantibody production in ectopic lymphoid tissue in the
chronic inflammatory disease Sjogren's syndrome.. Scand J Immunol.

[25] Xanthou G, Polihronis M, Tzioufas AG, Paikos S, Sideras P (2001). “Lymphoid” chemokine messenger RNA expression by
epithelial cells in the chronic inflammatory lesion of the salivary glands
of Sjogren's syndrome patients: possible participation in lymphoid
structure formation.. Arthritis Rheum.

[26] Carlsen HS, Baekkevold ES, Johansen FE, Haraldsen G, Brandtzaeg P (2002). B cell attracting chemokine 1 (CXCL13) and its receptor CXCR5 are
expressed in normal and aberrant gut associated lymphoid
tissue.. Gut.

[27] Festa ED, Hankiewicz K, Kim S, Skurnick J, Wolansky LJ (2009). Serum levels of CXCL13 are elevated in active multiple
sclerosis..

[28] Dobner T, Wolf I, Emrich T, Lipp M (1992). Differentiation-specific expression of a novel G protein-coupled
receptor from Burkitt's lymphoma.. Eur J Immunol.

[29] Meijer J, Zeelenberg IS, Sipos B, Roos E (2006). The CXCR5 chemokine receptor is expressed by carcinoma cells and
promotes growth of colon carcinoma in the liver.. Cancer Res.

[30] Singh S, Singh R, Singh UP, Rai SN, Novakovic KR (2009). Clinical and biological significance of CXCR5 expressed by
prostate cancer specimens and cell lines.. Int J Cancer.

[31] Tyagi SC (1998). Extracellular matrix dynamics in heart failure: a prospect for
gene therapy.. J Cell Biochem.

[32] Djebbari A, Quackenbush J (2008). Seeded Bayesian Networks: constructing genetic networks from
microarray data.. BMC Syst Biol.

[33] Hocking AM, Shinomura T, McQuillan DJ (1998). Leucine-rich repeat glycoproteins of the extracellular
matrix.. Matrix Biol.

[34] Svensson L, Aszodi A, Reinholt FP, Fassler R, Heinegard D (1999). Fibromodulin-null mice have abnormal collagen fibrils, tissue
organization, and altered lumican deposition in tendon.. J Biol Chem.

[35] Danielson KG, Baribault H, Holmes DF, Graham H, Kadler KE (1997). Targeted disruption of decorin leads to abnormal collagen fibril
morphology and skin fragility.. J Cell Biol.

[36] Hu C, Xiong J, Zhang L, Huang B, Zhang Q (2004). PEG10 activation by co-stimulation of CXCR5 and CCR7 essentially
contributes to resistance to apoptosis in CD19+CD34+ B cells from
patients with B cell lineage acute and chronic lymphocytic
leukemia.. Cell Mol Immunol.

[37] Chunsong H, Yuling H, Li W, Jie X, Gang Z (2006). CXC chemokine ligand 13 and CC chemokine ligand 19 cooperatively
render resistance to apoptosis in B cell lineage acute and chronic
lymphocytic leukemia CD23+CD5+ B cells.. J Immunol.

[38] Legler DF, Loetscher M, Roos RS, Clark-Lewis I, Baggiolini M (1998). B cell-attracting chemokine 1, a human CXC chemokine expressed in
lymphoid tissues, selectively attracts B lymphocytes via
BLR1/CXCR5.. J Exp Med.

[39] Moser B, Schaerli P, Loetscher P (2002). CXCR5(+) T cells: follicular homing takes center stage in
T-helper-cell responses.. Trends Immunol.

[40] Yamazaki T, Komuro I, Yazaki Y (1998). Signalling pathways for cardiac hypertrophy.. Cell Signal.

[41] Müller G, Lipp M (2001). Signal transduction by the chemokine receptor CXCR5: structural
requirements for G protein activation analyzed by chimeric CXCR1/CXCR5
molecules.. Biol Chem.

[42] Damås JK, Eiken HG, Oie E, Bjerkeli V, Yndestad A (2000). Myocardial expression of CC- and CXC-chemokines and their
receptors in human end-stage heart failure.. Cardiovasc Res.

[43] Dahl CP, Husberg C, Gullestad L, Waehre A, Damås JK (2009). Increased production of CXCL16 in experimental and clinical heart
failure: a possible role in extracellular matrix remodeling.. Circ Heart Fail.

[44] Monfort J, Tardif G, Reboul P, Mineau F, Roughley P (2006). Degradation of small leucine-rich repeat proteoglycans by matrix
metalloprotease-13: identification of a new biglycan cleavage
site.. Arthritis Res Ther.

[45] Morimoto H, Takahashi M, Izawa A, Ise H, Hongo M (2006). Cardiac overexpression of monocyte chemoattractant protein-1 in
transgenic mice prevents cardiac dysfunction and remodeling after myocardial
infarction.. Circ Res.

[46] Tachibana K, Hirota S, Iizasa H, Yoshida H, Kawabata K (1998). The chemokine receptor CXCR4 is essential for vascularization of
the gastrointestinal tract.. Nature.

[47] Doi M, Kusachi S, Murakami T, Ninomiya Y, Murakami M (2000). Time-dependent changes of decorin in the infarct zone after
experimentally induced myocardial infarction in rats: comparison with
biglycan.. Pathol Res Pract.

[48] Yamamoto K, Kusachi S, Ninomiya Y, Murakami M, Doi M (1998). Increase in the expression of biglycan mRNA expression
Co-localized closely with that of type I collagen mRNA in the infarct zone
after experimentally-induced myocardial infarction in rats.. J Mol Cell Cardiol.

[49] Weis SM, Zimmerman SD, Shah M, Covell JW, Omens JH (2005). A role for decorin in the remodeling of myocardial
infarction.. Matrix Biol.

[50] Westermann D, Mersmann J, Melchior A, Freudenberger T, Petrik C (2008). Biglycan is required for adaptive remodeling after myocardial
infarction.. Circulation.

[51] Bueno OF, De Windt LJ, Tymitz KM, Witt SA, Kimball TR (2000). The MEK1-ERK1/2 signaling pathway promotes compensated cardiac
hypertrophy in transgenic mice.. EMBO J.

[52] Sanna B, Bueno OF, Dai YS, Wilkins BJ, Molkentin JD (2005). Direct and indirect interactions between calcineurin-NFAT and
MEK1-extracellular signal-regulated kinase 1/2 signaling pathways regulate
cardiac gene expression and cellular growth.. Mol Cell Biol.

[53] Ding B, Price RL, Borg TK, Weinberg EO, Halloran PF (1999). Pressure overload induces severe hypertrophy in mice treated with
cyclosporine, an inhibitor of calcineurin.. Circ Res.

[54] Sjaastad I, Sejersted OM, Ilebekk A, Bjørnerheim R (2000). Echocardiographic criteria for detection of postinfarction
congestive heart failure in rats.. J Appl Physiol.

[55] Finsen AV, Christensen G, Sjaastad I (2005). Echocardiographic parameters discriminating myocardial infarction
with pulmonary congestion from myocardial infarction without congestion in
the mouse.. J Appl Physiol.

[56] Vinge LE, von Lueder TG, Aasum E, Qvigstad E, Gravning JA (2008). Cardiac-restricted expression of the carboxyl-terminal fragment
of GRK3 uncovers distinct functions of GRK3 in regulation of cardiac
contractility and growth: GRK3 controls cardiac alpha1-adrenergic receptor
responsiveness.. J Biol Chem.

[57] Liu H, Sanuda-Pena MC, Harvey-White JD, Kalra S, Cohen SA (1998). Determination of submicromolar concentrations of neurotransmitter
amino acids by fluorescence detection using a modification of the
6-aminoquinolyl-N-hydroxysuccinimidyl carbamate method for amino acid
analysis.. J Chromatogr A.

[58] Laurent GJ, Cockerill P, McAnulty RJ, Hastings JR (1981). A simplified method for quantitation of the relative amounts of
type I and type III collagen in small tissue samples.. Anal Biochem.

[59] Irizarry RA, Hobbs B, Collin F, Beazer-Barclay YD, Antonellis KJ (2003). Exploration, normalization, and summaries of high density
oligonucleotide array probe level data.. Biostatistics.

[60] Tusher VG, Tibshirani R, Chu G (2001). Significance analysis of microarrays applied to the ionizing
radiation response.. Proc Natl Acad Sci U S A.

[61] Finsen AV, Woldbæk PR, Li J, Wu J, Lyberg T (2004). Increased syndecan expression following myocardial infarction
indicates a role in cardiac remodeling.. Physiol Genomics.

[62] Calvert JW, Zhou C, Nanda A, Zhang JH (2003). Effect of hyperbaric oxygen on apoptosis in neonatal
hypoxia-ischemia rat model.. J Appl Physiol.

