# Supplementary figures and images for: Lack of Chemokine Signaling through CXCR5 Causes Increased Mortality, Ventricular Dilatation and Deranged Matrix during Cardiac Pressure Overload
Source: PLoS One. 2011 Apr 18;6(4):e18668. doi: 10.1371/journal.pone.0018668 (PMC3078912; doi:10.1371/journal.pone.0018668)

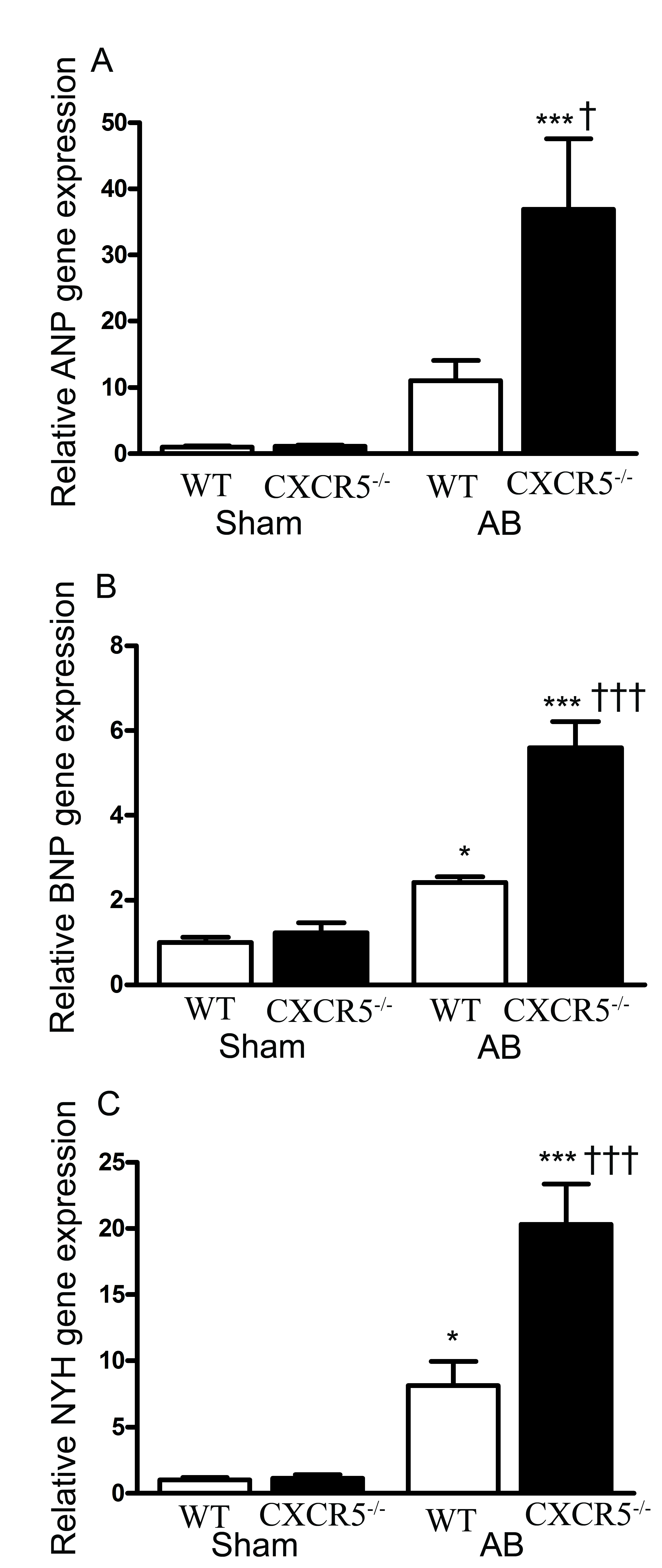

Supplement: Figure S1 — Altered expression of markers of cardiac wall stress and remodeling. Relative gene expression of (A) atrial natriuretic peptide (ANP), (B) brain natriuretic peptide (BNP) and (C) β-myosin heavy chain (MHC) in wild type (WT) Sham (n = 6), CXCR5-/- Sham (n = 6), WT aorta banded (AB) (n = 6), and CXCR5-/- AB (n = 6) groups. The results are mean ± SEM. *p<0.05 and ***p<0.001 vs. Sham groups; †p<0.05 and †††p <0.001 vs. WT AB group. (TIF) [file pone.0018668.s001.tif]

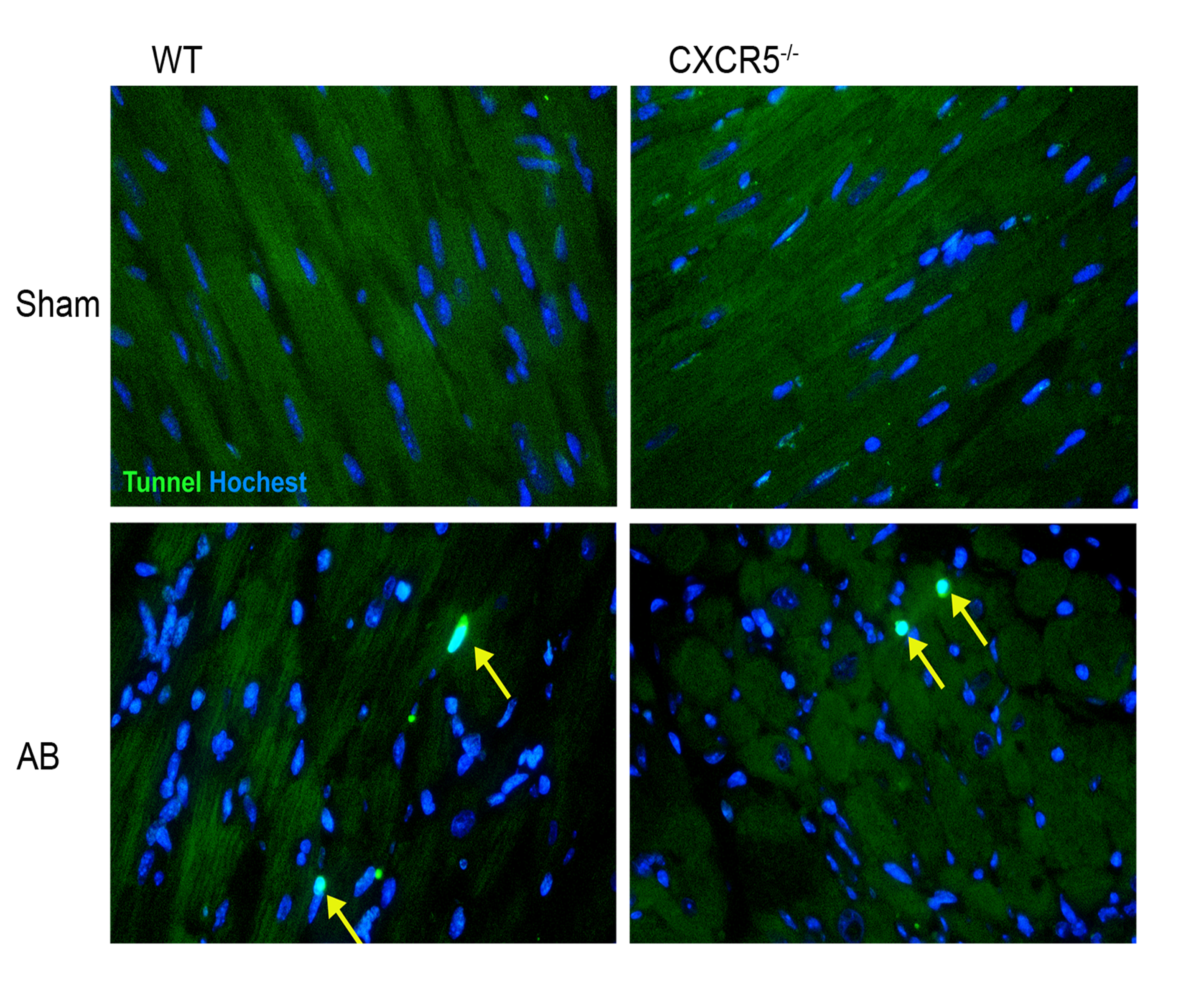

Supplement: Figure S2 — Fluorescent micrographs of sections of left ventricular myocardium from wild type (WT) and CXCR5-/- mice. The arrows indicate TUNEL-positive myocyte nucleus. (TIF) [file pone.0018668.s002.tif]

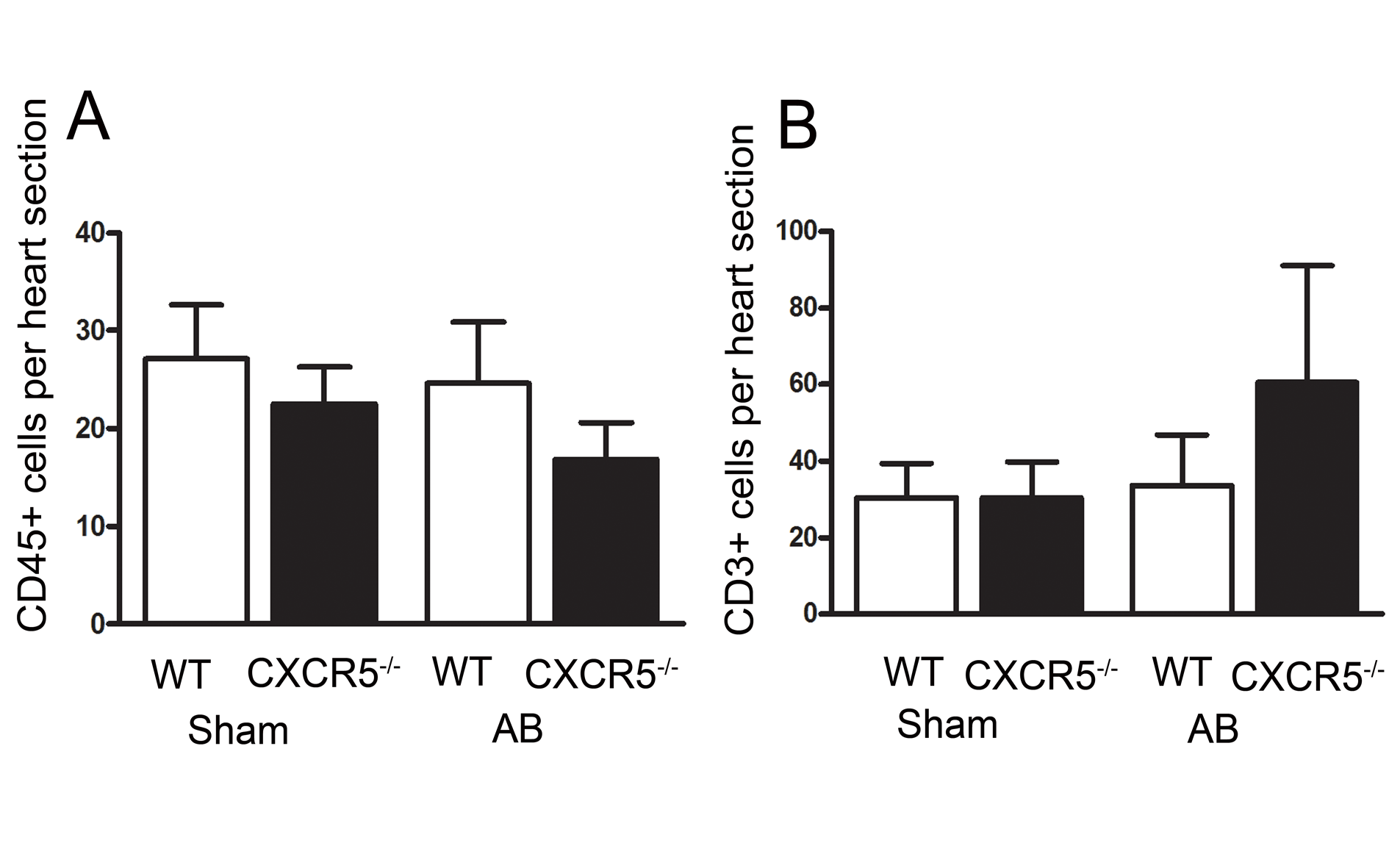

Supplement: Figure S3 — CD45 and CD3 positive lymphocytes in the left ventricular myocardium from wild type (WT) and CXCR5-/- mice. Total number of CD45R (A) and CD3 (B) postive lymphocytes was not significantly different between CXCR5-/- and WT mice after sham operation or AB. (n = 6 heart sections in all groups). Cells counted from 32 digital, evenly distributed images (x400) from each heart.The results are mean ± SEM. (TIF) [file pone.0018668.s003.tif]
