# Supplementary material for: Lack of Chemokine Signaling through CXCR5 Causes Increased Mortality, Ventricular Dilatation and Deranged Matrix during Cardiac Pressure Overload
Source: PLoS One. 2011 Apr 18;6(4):e18668. doi: 10.1371/journal.pone.0018668 (PMC3078912; doi:10.1371/journal.pone.0018668)
Supplement: Table S1 — Weights and echocardiographic measurements. Values are means ± SE. BW, body weight; TL, tibia length; LVW, left ventricular weight; LW, lung weight; IVSd and IVSs, interventricular septum thickness in diastole and in systole, respectively; LVDd and LVDs, left ventricular diameter in diastole and in systole, respectively; FS, fractional shortening in LVD; LVPWd and LVPWs, posterior wall thickness in diastole and in systole, respectively; LAD, left atrial diameter; HR, heart rate; AVmax, peak aortic stenosis flow velocity; TVs, peak tissue velocity in systole; TVd, peak tissue velocity in diastole. LVW/TL and LW/TL are mg/mm. * p<0.05, ** p<0.01, *** p<0.001 vs. Non-operated and Sham groups. †p<0.05, ††, p <0.01, †††p<0.001 vs. WT AB group. ‡p<0.05 vs CXCR5-/- Non-operated group. The results are mean ± SEM. (DOC) [file pone.0018668.s004.doc]

**Table S1. Weights and echocardiographic measurements**

|  | WT | CXCR5^-/-^ | WT Sham | CXCR5^-/-^ Sham | WT AB | CXCR5^-/-^ AB |
| --- | --- | --- | --- | --- | --- | --- |
| *n* | 6 | 6 | 7 | 7 | 6 | 8 |
| *Weights* |  |  |  |  |  |  |
| BW (g) | 26.2±0.8 | 25.3±0.7 | 27.9±0.9 | 25.8±0.9 | 27.8±1.0 | 26.4±0.6 |
| TL (mm) | 18.2±0.1 | 17.3±0.3 | 18.0±0.1 | 17.6±0.1 | 18.3±0.1 | 17.3±0.4 |
| LVW/TL | 5.4±0.2 | 5.2±0.3 | 5.5±0.2 | 5.3±0.3 | 8.6±0.5*** | 8.6±0.3** |
| LW/TL | 8.9±0.2 | 9.0±0.5 | 8.7±0.2 | 9.1±0.5 | 17.6±2.5** | 16.0±2.7* |
| *Echocardiography* |  |  |  |  |  |  |
| *M-Mode* |  |  |  |  |  |  |
| IVSd (mm) | 0.56±0.09 | 0.56±0.06 | 0.68±0.07 | 0.62±0.03 | 0.98±0.18** | 0.67±0.12† |
| IVSs (mm) | 0.87±0.08 | 0.83±0.15 | 1.02±0.13 | 0.96±0.17 | 1.38±0.27*** | 0.79±0.16††† |
| LVIDd (mm) | 4.50±0.37 | 4.28±0.17 | 4.43±0.30 | 4.59±0.32 | 4.31±0.29 | 5.40±0.59*† |
| LVIDs (mm) | 3.63±0.42 | 3.42±0.22 | 3.54±0.56 | 3.68±0.25 | 3.43±0.47 | 5.03±0.80**†† |
| FS (%) | 19.13±4.55 | 20.25±4.07 | 20.86±2.34 | 19.67±1.03 | 20.50±5.75 | 7.50±5.21*† |
| LVPWd (mm) | 0.63±0.09 | 0.61±0.06 | 0.67±0.15 | 0.60±0.11 | 1.10±0.22*** | 0.68±0.14††† |
| LVPWs (mm) | 0.84±0.09 | 0.76±0.10 | 0.98±0.19 | 0.79±0.07 | 1.36±0.16*** | 0.87±0.22††† |
| LAD (mm) | 1.59±0.2 | 1.78±0.14 | 1.73±0.14 | 1.71±0.09 | 2.58±0.55*** | 3.17±0.41*** |
| *Doppler* |  |  |  |  |  |  |
| HR (BMP) | 430.3±78.03 | 471.8±36.3 | 417.6±29.2 | 433.3±58.4 | 453.8±92.7 | 463.5±68.9 |
| AVmax (m/s) |  |  |  |  | 4.12±0.19 | 4.33±0.54 |
| TVs (cm/s) | 1.33±0.37 | 1.54±0.30 | 1.18±0.35 | 1.11±0.16‡ | 0.77±0.28*** | 0.66±0.31*** |
| TVd (cm/s) | 1.50±0.27 | 1.63±0.19 | 1.18±0.60 | 0.99±0.18 | 0.91±0.47 | 0.73±0.38** |
