# Supplementary material for: Lack of Chemokine Signaling through CXCR5 Causes Increased Mortality, Ventricular Dilatation and Deranged Matrix during Cardiac Pressure Overload
Source: PLoS One. 2011 Apr 18;6(4):e18668. doi: 10.1371/journal.pone.0018668 (PMC3078912; doi:10.1371/journal.pone.0018668)
Supplement: Table S2 — Characteristics of the real-time PCR assays used in the human study. The table shows the sequence of primers used in the real-time PCR assays. (+), forward primers; (–), reverse primers; Acc.nr, GenBank accession number; GAPDH, glyceraldehyde 3-phosphate dehydrogenase. (DOC) [file pone.0018668.s005.doc]

Table S2. Real-time PCR assays in the human study

| Target | Sequence (5’🡪3’) | Acc.Nr. |
| --- | --- | --- |
| Biglycan | (+)-GAATGAACTCCACCTAGACCACAAC | NM_001711 |
|  | (–)-AGGCCCAGCCTGTACAGCTT |  |
| CXCR5 | (+)-GCCGGCACAGCCATGA | NM_001716 |
|  | (–)-CTGTCCAGTTCCCAGAACAGGT |  |
| Fibromodulin | (+)-CTCTCCCAGGCCCAGTATGA | NM_002023 |
|  | (–)-TAAGGGTCATAGGGATCGTAGTAGGT |  |
| GAPDH | (+)-CCAAGGTCATCCATGACAACTT | NM_002046 |
|  | (–)-AGGGGCCATCCACAGTCTT |  |
| Lumican | (+)-GGGCAATCATCACCAAACTGT | NM_002345 |
|  | (–)-AGGAGGCACCATTGGTACACTT |  |
